# Supplementary material for: Twin-arginine translocase component TatB performs folding quality control via a chaperone-like activity
Source: Sci Rep. 2022 Sep 1;12:14862. doi: 10.1038/s41598-022-18958-3 (PMC9436932; doi:10.1038/s41598-022-18958-3)
Supplement: Supplementary file 1 — Supplementary Information. [file 41598_2022_18958_MOESM1_ESM.pdf]

Supplementary Information

Twin-arginine translocase component TatB performs folding quality control via a chaperone-like activity

May N. Taw[1], Jason T. Boock[2], Daniel Kim[2], Mark A. Rocco[3], Dujduan Waraho-Zhmayev[4] and Matthew P. DeLisa[1,2,3]*

[1]Department of Microbiology, Cornell University, Ithaca, NY 14853 USA
[2]Robert F. Smith School of Chemical and Biomolecular Engineering, Cornell University, 120 Olin Hall, Ithaca, NY 14853 USA
[3]Nancy E. and Peter C. Meinig School of Biomedical Engineering, Cornell University, Ithaca, NY 14853 USA
[4]Biological Engineering, Faculty of Engineering, King Mongkut's University of Technology Thonburi, Bangkok, Thailand

Supplementary Table 1. Bacterial strains and plasmids used in this study

**Bacterial strain**	**Genotype**	**Source**
MC4100	F$^-$ *araD139* Δ(*argF-lac*)*169* λ$^-$ *e14*$^-$ *flhD5301* Δ(*fruK-yeiR*)*725(fruA25)* *relA1 rpsL150*(Str^R) *rbsR22* Δ(*fimB*-fimE)632(::IS*1*) *deoC1*	(1)
DADE	MC4100 Δ*tatABCD*Δ*tatE*	(2)
BL21(DE3)	F$^-$ *ompT hsdS*$_B$ (r_B^-, m_B^-) *gal dcm* (DE3)	Laboratory stock
MC1000	F′ [*proAB lacI*^{Q} *lacZ* ΔM15 Tn*10*(Tet^R)] *araD139* Δ(*araA-leu*)7679 (*codB-lac*)X74 *galE15 galK16 rpsL150 relA1 thi*	
DHB4	MC1000 Δ*phoA*(*Pvu*II) *phoR* Δ*malF3*	(3)
DR473	DHB4 Δ*trxB gor552* Tn*10*(Tet^R) *ahpC** Tn*10*(Cm^R) (*araC* P_{ara}-*trxB*)	(4)
MCMTA	MC4100 *tatB*::kan	(5)
DRB	DR473 *tatB*::kan	(3)
**Bacterial plasmids**		
pTatABC	Entire *E. coli tatABC* operon including native promoter in pBR322; Tet^R	(6)
pTatABC-XX	pTatABC with XbaI and XhoI restriction sites flanking *tatB* and *tatC* genes, respectively	(6)
pTatAC	pBR322 with *tatAC* cloned between PvuI and AhdI	This work
pTatAB(-10)C-XX and related TatB truncation plasmids	pTatABC-XX with truncation of *tatB* at 3' end by 10, 20, 30, 40, 50, 60, 70, 80, 90, 100, 110, 120, 130, and 140 codons	This work
pTatAB(H109A/H112A/ H123A)C-XX	pTatABC-XX with single or triple His to Ala mutation (H109A, H112A, or H123A) in TatB	This work
pTorA-AP	Signal peptide derived from *E. coli* trimethylamine *N*-oxide reductase (TorA) fused to gene encoding mature *E. coli* alkaline phosphatase (APΔ1-22) cloned in plasmid pTrc99A	(3)
pET22-TatB$^{22\text{-}171}$	*E. coli tatB* gene lacking N-terminal TMH (residues 1-21) cloned with polyhistidine (6x-His) tag in plasmid pET-22b(+)	This work
pET22-YpTatB$^{22\text{-}220}$	*Y. pseudotuberculosis tatB* gene lacking N-terminal TMH (residues 1-21) cloned with polyhistidine (6x-His) tag in plasmid pET-22b(+)	This work
pET22-TatB$^{22\text{-}91}$	*E. coli tatB* gene lacking N-terminal TMH and truncated C-terminally to terminate at residue 91 with 6x-His tag in pET-22b(+)	This work
pET22-YpTatB$^{22\text{-}91}$	*Y. pseudotuberculosis tatB* gene lacking N-terminal TMH and truncated C-terminally to terminate at residue 91 with 6x-His tag in pET-22b(+)	This work
pSALect-α3 plasmids	Codon-optimized genes encoding α3A/B/C/D proteins cloned between spTorA and Bla in plasmid pSALect	(6)
pTatA(YpB)C	Gene encoding *Y. pseudotuberculosis* TatB replacing *E. coli* TatB in plasmid pTatABC-XX	This work
pTatA(YpB$^{1\text{-}91}$)C	Gene encoding C-terminally truncated *Y. pseudotuberculosis* TatB (residues 1-91) replacing *E. coli* TatB in plasmid pTatABC-XX	This work
pMAF10-TatB	Gene encoding *E. coli* TatB cloned between EcoRI and XbaI in plasmid pMAF10	This work
pMAF10-YpTatB	Gene encoding *Y. pseudotuberculosis* TatB cloned between EcoRI and XhoI in plasmid pMAF10	This work
pTatABC-XX-3x-FLAG	pTatABC-XX with 3x-FLAG tag fused to C-terminus of TatC	This work
pTatAB$^{1\text{-}91}$C-XX-3x-FLAG	pTatABC-XX with truncated TatB (residues 1-91) and 3x-FLAG tag fused to C-terminus of TatC	This work
pTatAB(H109A/H112A/ H123A)C-XX-3x-FLAG	pTatABC-XX with single or triple His to Ala mutation (H109A, H112A, or H123A) in TatB and 3x-FLAG tag fused to C-terminus of TatC	This work

References cited

1. E. G. Bogsch *et al.* (1998) An essential component of a novel bacterial protein export system with homologues in plastids and mitochondria. *J Biol Chem* 273, 18003-18006.
2. M. Wexler *et al.* (2000) TatD is a cytoplasmic protein with DNase activity - No requirement for TatD family proteins in Sec-independent protein export. *J Biol Chem* 275, 16717-16722.

3. M. P. DeLisa, D. Tullman, G. Georgiou (2003) Folding quality control in the export of proteins by the bacterial twin-arginine translocation pathway. *Proc Natl Acad Sci USA* 100, 6115-6120.
4. P. H. Bessette, F. Aslund, J. Beckwith, G. Georgiou (1999) Efficient folding of proteins with multiple disulfide bonds in the *Escherichia coli* cytoplasm. *Proc Natl Acad Sci U S A* 96, 13703-13708.
5. A. Chanal, C. L. Santini, L. F. Wu (1998) Potential receptor function of three homologous components, TatA, TatB and TatE, of the twin-arginine signal sequence-dependent metalloenzyme translocation pathway in *Escherichia coli*. *Mol Microbiol* 30, 674-676.
6. M. A. Rocco, D. Waraho-Zhmayev, M. P. DeLisa (2012) Twin-arginine translocase mutations that suppress folding quality control and permit export of misfolded substrate proteins. *Proc Natl Acad Sci USA* 109, 13392-13397.

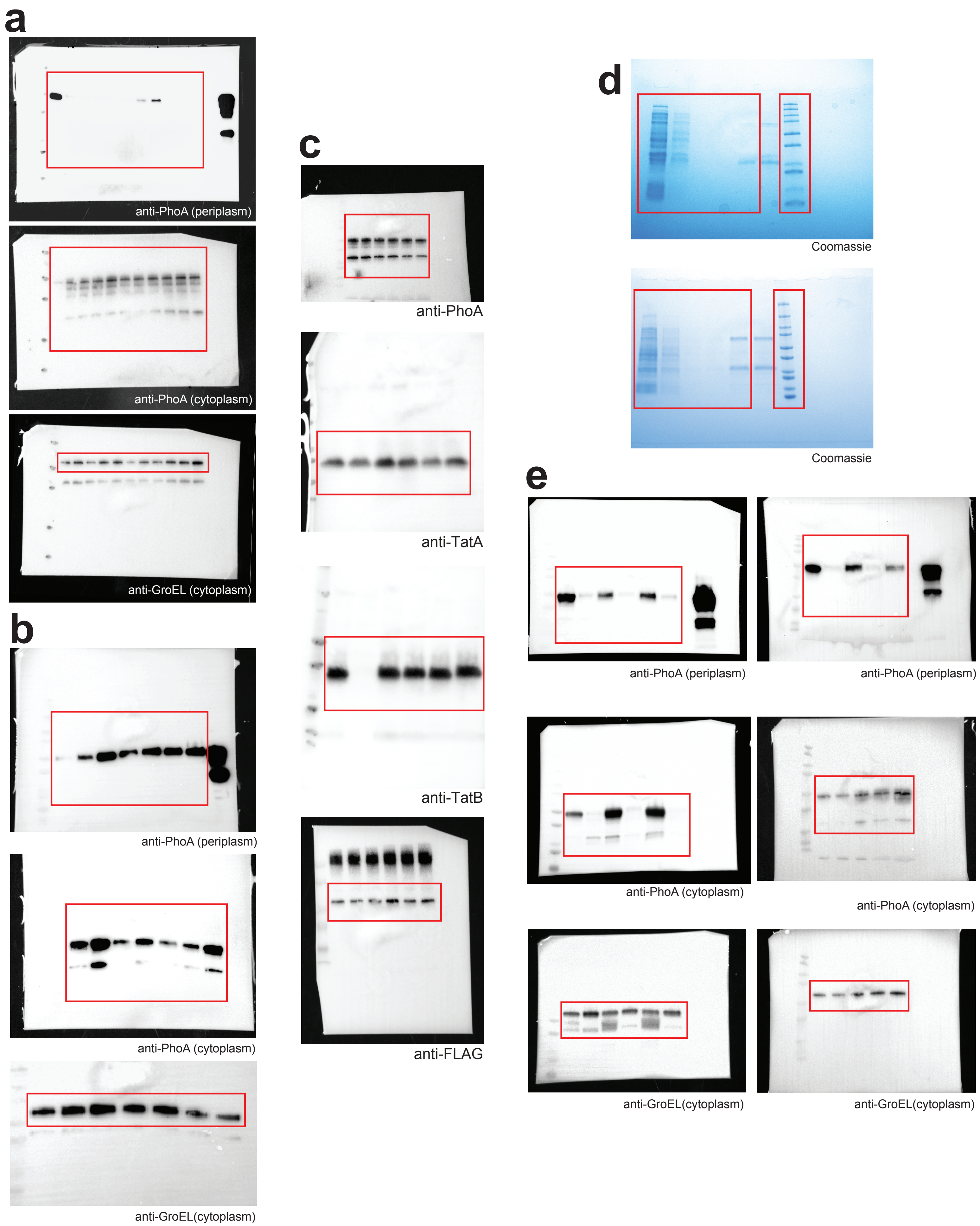

Supplementary Figure 1. Source Data for main and supplementary figures. Uncropped, unprocessed images corresponding to immunoblots or SDS-PAGE gels presented in: (a) Figure 2c; (b) Figure 3c; (c) Supplementary Figure 2; (d) Supplementary Figure 4b; and (e) Supplementary Figure 5a. Red boxes depict cropped regions.

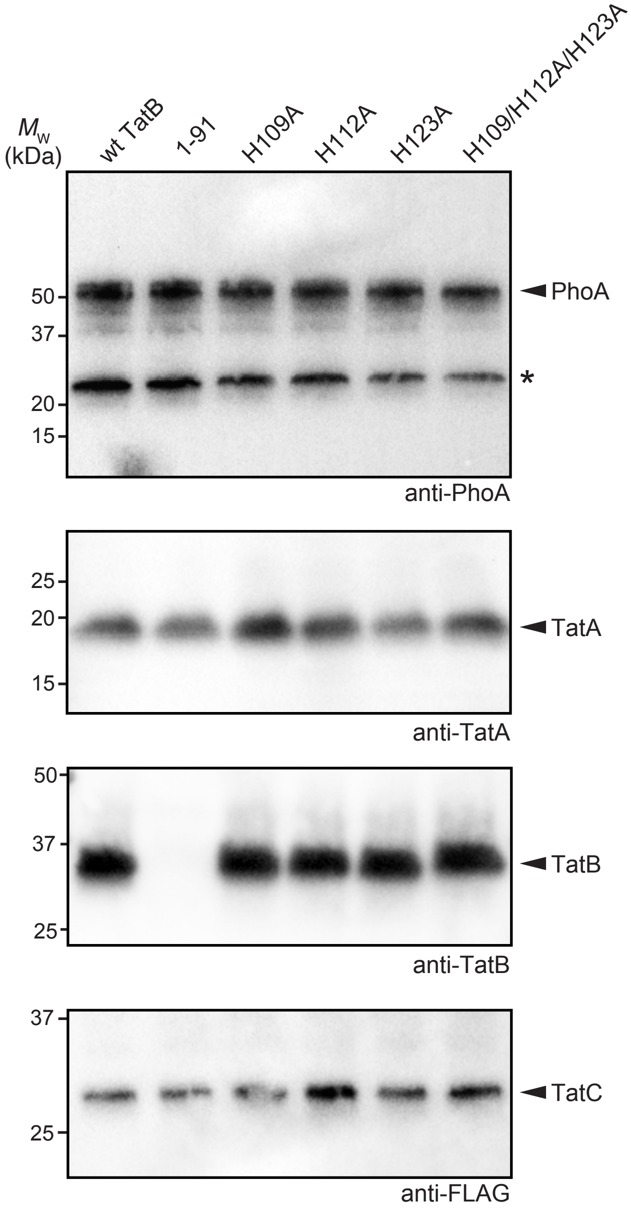

Supplementary Figure 2. Expression analysis of PhoA and TatABC proteins. Western blot analysis of soluble and membrane fractions prepared from DADE cells co-expressing Tat-targeted PhoA from pTor-AP along with a Tat operon plasmid encoding either wt or one of the TatB variants as indicated. Total PhoA expression levels were determined by collecting the soluble fraction from an equivalent number of cells and probing the membrane with anti-PhoA antibody. Total TatA, TatB, and TatC expression levels were determined by collecting the membrane fraction from an equivalent number of cells and probing with serum against TatA or TatB to detect TatA and TatB proteins, respectively, or an anti-FLAG antibody to detect 3x-FLAG tag introduced at the C-terminus of TatC. TatB1-91 was not detected by the anti-TatB serum due to the truncation of a significant portion of TatB. Asterisk indicates spTorA-PhoA breakdown product. Molecular weight (MW) markers are indicated on the left. Results are representative of three biological replicates. See Supplementary Figure 1 for uncropped versions of the images.

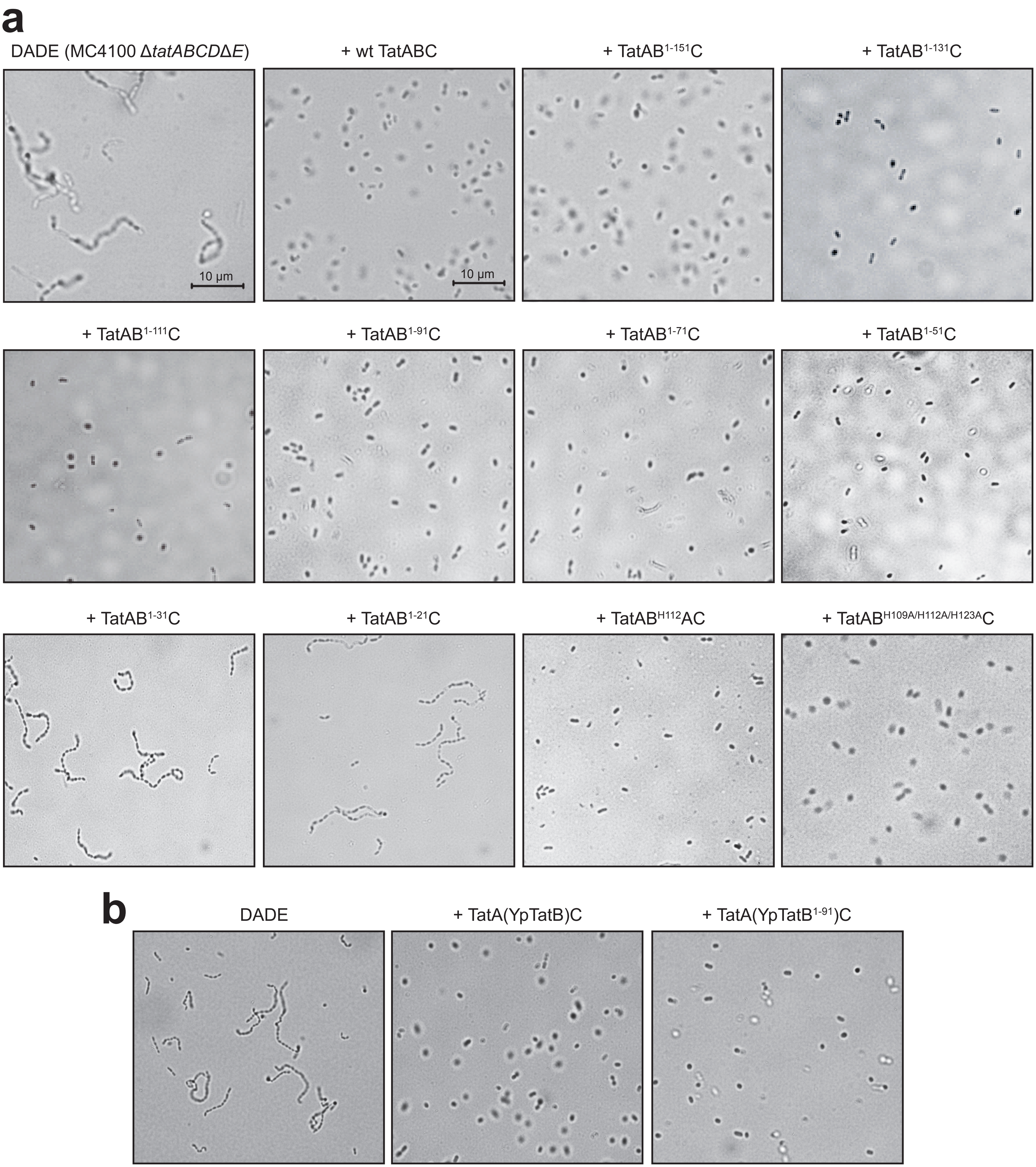

Supplementary Figure 3. Growth phenotypes conferred by TatB mutants. (a) Light microscopy of plasmid-free DADE cells or DADE cells carrying pTatABC-XX-based plasmids expressing either wt TatABC or mutant translocases in which TatB was truncated or carrying substitutions in the essential histidine residues as indicated. (b) Light microscopy of plasmid-free DADE cells or DADE cells carrying pTatABC-XX-based plasmids expressing wt TatAC along with either wt YpTatB or truncated YpTatB$^{1-91}$.

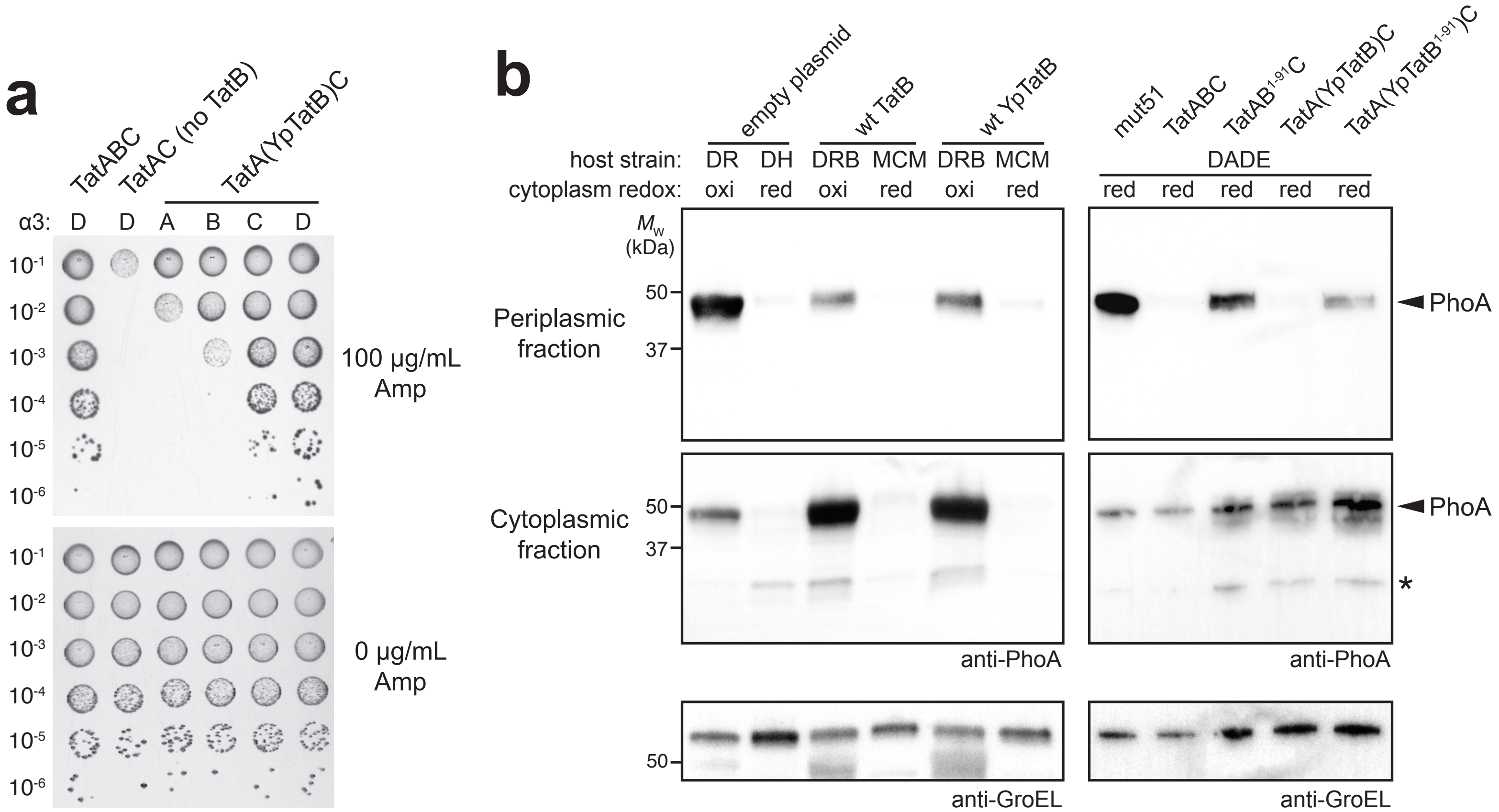

Supplementary Figure 4. Regulation of QC activity by YpTatB in *E. coli*. (a) Resistance of serially diluted DADE cells co-expressing one of the spTorA-α3-Bla chimeras (A, B, C or D) along with a Tat operon plasmid encoding wt TatABC or TatAC from *E. coli* without TatB or with YpTatB. Cells were spotted on LB-agar plates containing either 100 µg/mL Amp or 25 µg/mL chloramphenicol (0 µg/mL Amp). (b) Western blot analysis of cytoplasmic (cyt) and periplasmic (per) fractions prepared from: (left panel) DRB cells with oxidizing cytoplasm (DRB, oxi) or MCMTA cells with reducing cytoplasm (MCM, red) co-expressing Tat-targeted PhoA from pTorA-AP along with either TatB from plasmid pMAF10-TatB or YpTatB from plasmid pMAF10-YpTatB; and (right panel) DADE cells with reducing cytoplasm carrying pTatABC-XX-based plasmids encoding copies of wt TatABC or translocases with $TatB^{1\text{-}91}$, YpTatB, or $YpTatB^{1\text{-}91}$ as indicated. DR473 (DR, oxi) and DHB4 (DH, red) cells carrying empty pMAF10 plasmid served as positive and negative controls, respectively. DADE cells carrying a plasmid encoding QCS suppressor mut51 were also included as a positive control. An equivalent number of cells was loaded in each lane. PhoA was probed with anti-PhoA antibody while anti-GroEL antibody confirmed equivalent loading in each lane. Asterisk indicates spTorA-PhoA breakdown product. Molecular weight (MW) markers are indicated on the left. Results are representative of three biological replicates. See Supplementary Figure 1 for uncropped versions of the images.

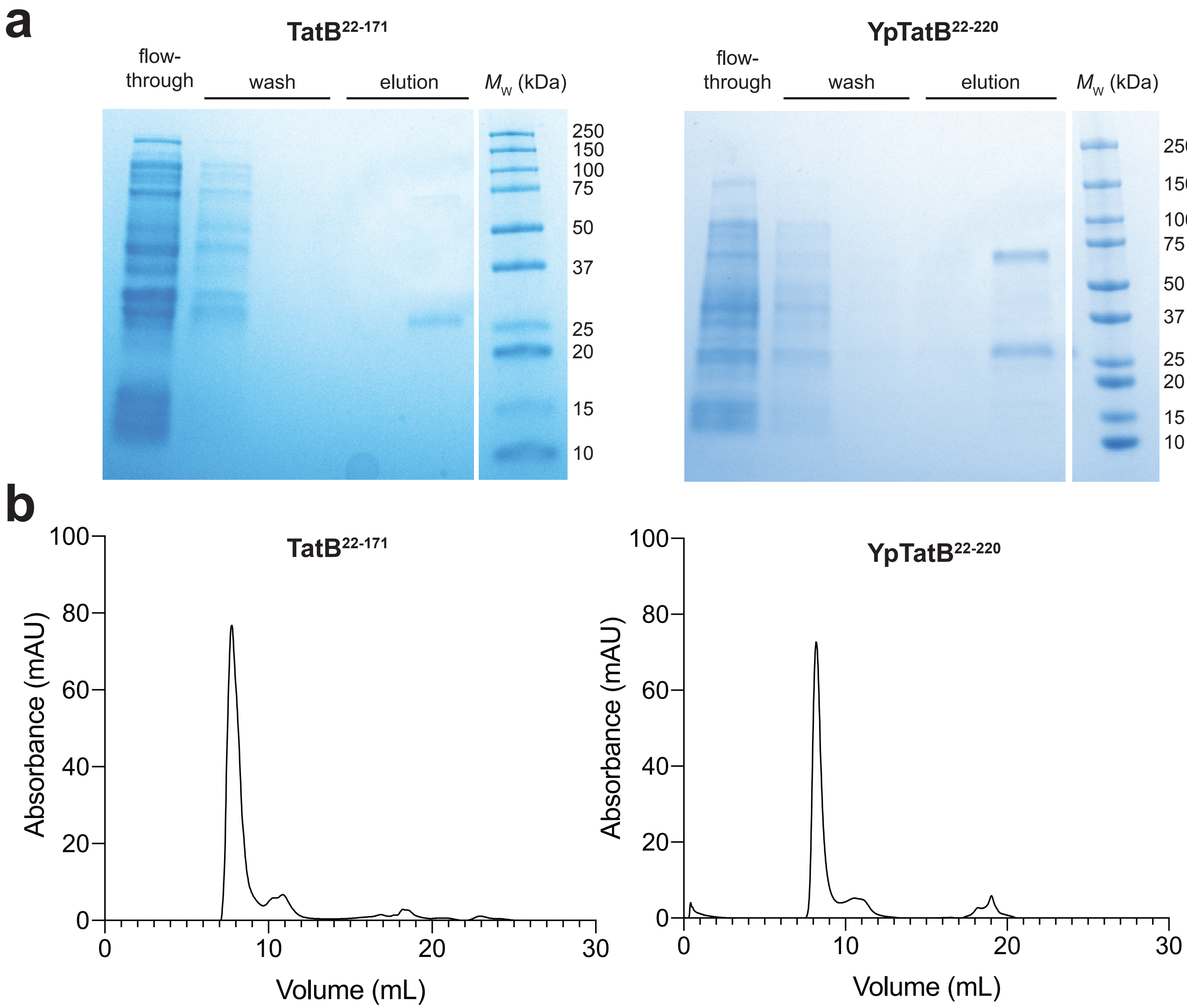

Supplementary Figure 5. Purification of TatB and YpTatB proteins. (a) Coomassie blue-stained SDS-PAGE gels showing various fractions of $TatB^{22\text{-}171}$ (left panel) and $YpTatB^{22\text{-}220}$ (right panel) proteins as indicated. Molecular weight (M_w) ladders shown at right. Each ladder was run in same gel but image was cropped to remove additional lanes. Gels are representative of three biological replicates. See Supplementary Figure 1 for uncropped versions of the images. (b) SEC analysis of Ni-NTA-purified *E. coli* $TatB^{22\text{-}171}$ and *Y. pseudotuberculosis* $TatB^{22\text{-}220}$ using GE Superdex 75 10/300 GL.

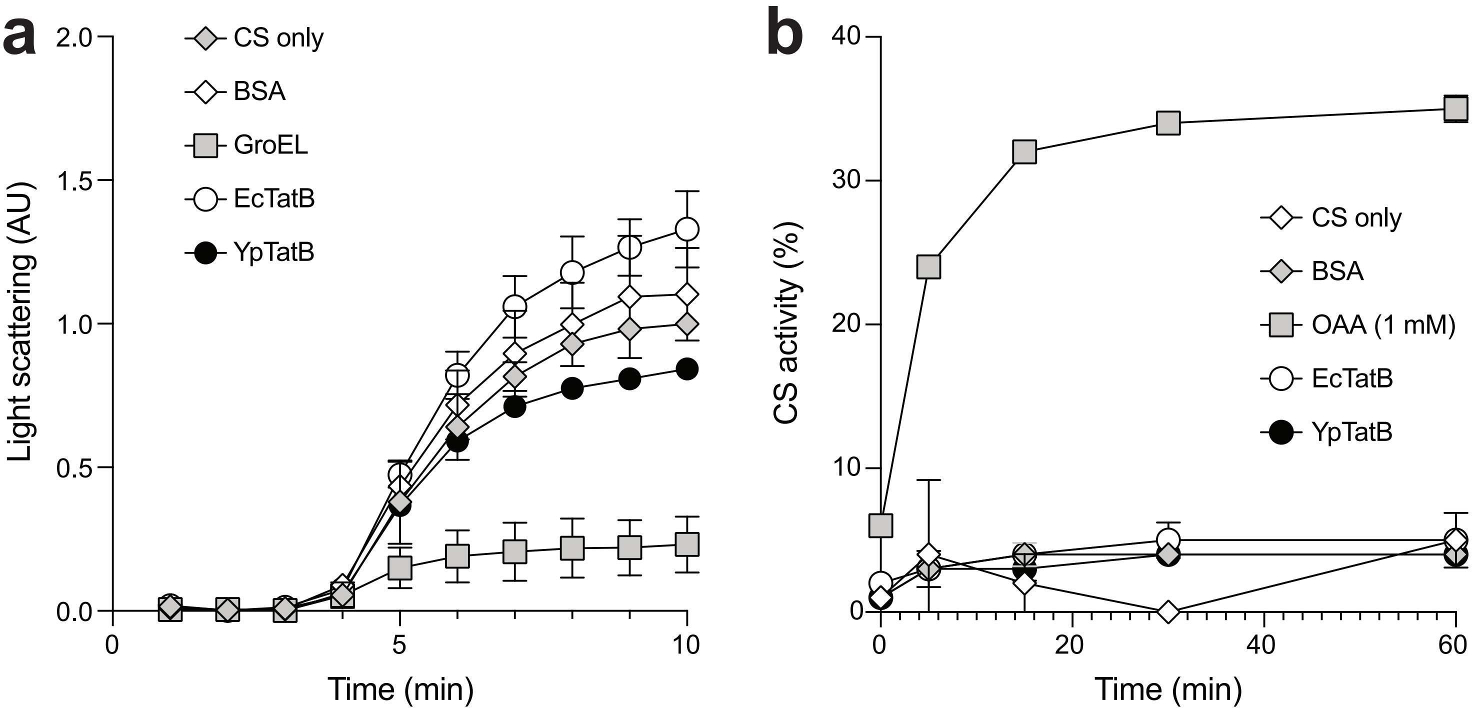

Supplementary Figure 6. Effect of TatB and YpTatB on aggregation of chemically denatured CS and reactivation of thermally denatured CS. (a) CS aggregation monitored by light scattering measurements over time without additional factors or in the presence of 0.15 µM of $TatB^{22\text{-}171}$, $YpTatB^{22\text{-}220}$, GroEL, or BSA. CS was chemically denatured in 6 M Gdn-HCl and diluted to a final concentration of 0.15 µM into 50 mM Tris-HCl, pH 8.0. (b) CS (0.15 µM) was inactivated at 43°C in the presence of 0.6 µM $TatB^{22\text{-}171}$, $YpTatB^{22\text{-}220}$, BSA, or 1 mM oxaloacetate (OAA) for 30 min prior to reactivation by transferring samples to room temperature. CS reactivation kinetics were monitored by measuring CS activity at different time points following inactivation. OAA is a substrate of CS and also a known 'stabilizer' for slowing down unfolding CS intermediates from irreversible aggregation.
